# Determination of squalene in edible oils by transmethylation and GC analysis

**DOI:** 10.1016/j.mex.2018.12.001

**Published:** 2018-12-04

**Authors:** Suzanne M. Budge, Christopher Barry

**Affiliations:** Department of Process Engineering and Applied Science, Dalhousie University, Halifax, NS, B3H 4R2, Canada

**Keywords:** Squalene determination by GCFID, Fatty acid methyl esters, Basic catalyst, 23:0, Internal standard

## Abstract

Squalene is a triterpenoid with medicinal, therapeutic and cosmetic applications. There is interest in identifying novel sources of squalene, creating a need for a rapid and accurate method of squalene determination. Here, we describe such an approach that involves first transmethylating the oil base containing squalene using a basic catalyst, followed by addition of internal standard and quantification by gas chromatography flame ionization detection (GCIFD).

• The method uses a single reference standard for quantification.
• Validation showed it to be both repeatable and accurate.
• It was successfully applied to nutraceutical and edible oils containing squalene.

## Graphical abstract

**Figure:**
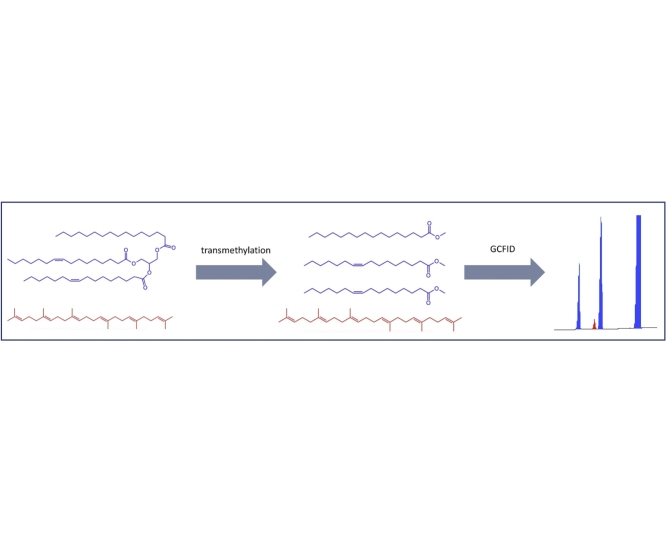

**Specifications Table**

**Table:** 

Subject area	• *Agricultural and Biological Sciences* • *Chemistry*
More specific subject area	*Analytical chemistry, bioactives*
Method name	*Squalene determination by GCFID*
Name and reference of original method	*R.G. Ackman, E.J. Macpherson, A. Timmins, Squalene in oils determined as squalane by gas-liquid chromatography after hydrogenation of methyl esters. J. Amer. Oil Chem. Soc. 77 (2000) 831-836.*
Resource availability	*NA*

## Method details

Squalene is a naturally occurring triterpenoid, serving as a biochemical precursor to sterols. It was first identified in shark liver oils [1] and has been shown to have significant medicinal, therapeutic and cosmetic applications [2,3]. More recently, it has been identified as a potential conjugate in drug design [4,5]. Such interest has prompted efforts to identify sources of squalene in addition to shark liver, with olive, amaranth and algal oils as potential sources, and highlighting the need for a rapid method for accurate determination of squalene.

As a non-polar triterpenoid, squalene is typically isolated from an oil base. Because the large concentration of lipids interferes with direct determination, a common route to its quantification has been saponification of the oil, followed by isolation of the non-saponifiable fraction that contains squalene and other non-acyl structures. Separation and quantification of the non-saponifiable fraction is then carried out by gas chromatography (GC) [6] or high performance liquid chromatography (HPLC) [7]. Alternatively, Grigoriadou et al. [8] used solid-phase extraction to isolate non-polar material including squalene. Non-chromatographic alternatives have also been suggested, including NMR [9] and Raman spectrometry [10]. However, all recent methods have overlooked the approach advocated by Ackman et al. [11], where acyl lipids were transmethylated to generate fatty acid methyl esters (FAME); squalene was recovered with FAME and all were hydrogenated to eliminate co-elution. Both FAME and squalene (as squalane) were then determined simultaneously by GC with flame ionization detection (FID) using an external standard method. Here, we describe improvements on this method by eliminating the hydrogenation step and using an internal standard.

We have based our internal standard calibration method on that recommended by the Global Organization for EPA and DHA [12] for analysis of long-chain omega-3 fatty acids. This method employs tricosanoic acid methyl ester (23:0) as an internal standard with a single point calibration conducted with each batch of samples. We combined this approach with a base-catalyzed procedure to generate methyl esters since acidic catalysts, particularly BCl_3_ in methanol, are known to decompose squalene [13]. Base-catalyzed transmethylation also offers the advantage of proceeding very rapidly (∼2 min.) at room temperature. We then analyzed squalene by GCFID using a column with a polar phase of (50% cyanopropyl)-methylpolysiloxane to avoid the co-elution issues identified by Ackman et al. [11].

### Materials

Olive oil (squalene at ∼1 mg g^−1^) was purchased from a local grocer, amaranth oil (∼50 mg g^−1^) from Fronaturals Canada and shark liver oil (∼250 mg g^−1^) from Bell Lifestyle Products. Fish oil was donated by Nature’s Way Canada; an algal oil (squalene at ∼5 mg g^−1^) was supplied by a collaborator. Squalene and 23:0 FAME were purchased from Sigma. All solvents were HPLC grade.

### General procedure for synthesis of FAME and isolation of squalene

Samples were dissolved in *t*-butyl methyl ether (*t*-BME) and known portions of each containing < 100 mg lipid were placed in a test tube, the volume was reduced to 1 ml using an N_2_ evaporator and 0.5 ml 0.2 M sodium methoxide in methanol was added. The mixture was shaken vigorously for 1 min, and then allowed to stand for 2 min at room temperature. To stop the reaction, 0.1 ml 0.5 M H_2_SO_4(aq)_ was added, and mixed vigorously for 15 s. Distilled water (∼1.5 ml) was then added, followed again with mixing for 15 s. The mixture was centrifuged at ∼ 200 g for 5 min and the upper phase containing FAME and squalene was collected and dried over anhydrous Na_2_SO_4_. The upper phase was then transferred to a final test tube, the solvent was evaporated and the sample was made up to 5 mg squalene ml^−1^ in hexane. Total time required for sample preparation was typically < 30 min.

### Calibration curve for validation of single-point internal standard method

A series of standard solutions of squalene in *t*-BME with concentrations ranging from ∼ 0.05–20 mg ml^−1^ were created by serial dilution. Each standard also contained 23:0 FAME, serving as internal standard, at a concentration of 1.0 mg ml^−1^. Aliquots of each standard, containing < 100 mg total mass and ranging from 2.5 to 10 ml, were carried through the FAME synthesis procedure and made up to ∼ 5 mg squalene ml^−1^ in hexane. The standard curve was prepared separately in triplicate to demonstrate the linear range of response relative to concentration.

### Sample preparation

Each oil sample (ranging from 0.3 to 2.0 g) was accurately weighed into a 25-ml volumetric flask and an appropriate mass of 23:0 FAME was added. When possible, the mass of 23:0 added was varied with the expected squalene content and known oil mass so that 23:0 would have a similar peak area as squalene. Samples were made up to volume with *t*-BME and well mixed. A known volume, containing < 100 mg lipid, and ranging from 1 to 6 ml, was carried through the FAME preparation method. Samples were made up to an expected squalene concentration of ∼5 mg ml^−1^ in hexane, and squalene was determined by reference to a single reference standard (below).

### Reference standards

Reference standards of squalene and 23:0 in *t*-BME, consisting of known and near equal masses of both in a 25-ml volumetric flask, were prepared. The concentration of squalene in the reference standard was chosen to be similar to that anticipated in the sample. A volume matching that taken from the sample was then removed and carried through the FAME preparation. Final squalene concentrations were adjusted to ∼5 mg ml^−1^ in hexane.

### Test of trueness

Certified reference materials were not available for squalene so spiking and recovery tests were carried out as an alternative method to access trueness. Three batches of fish oil that did not contain squalene were spiked with known amounts of squalene to match the range of concentrations anticipated in the oil samples. Three aliquots of each spiked fish oil were then carried through the same sample preparation method for synthesis of FAME with addition of 23:0 FAME at similar concentration to squalene and made up to 5 mg squalene ml^−1^ in hexane. Reference standards with known masses of 23:0 and squalene, at concentrations similar to those of the spiked fish oil, were made and an aliquot of each was also carried through the FAME preparation as described above.

### Test of repeatability

Five replicates of each sample of olive, shark liver, amaranth and algal oil were carried through the same sample preparation method with 23:0 FAME added to each sample at concentrations similar to the expected concentration of squalene. A representative reference standard was prepared for each sample to match the range of squalene concentrations expected in the oil and was carried though the FAME procedure.

### GC conditions

All samples and standards were analyzed by GCFID. FAME and squalene were separated using a DB-23 column ((50% cyanopropyl)-methylpolysiloxane, 30 m, 0.25 mm ID, 0.25 um d_f_) with He as carrier gas at a flow rate of 0.8 ml min^−1^. The column temperature was held initially at 150 °C and was then ramped at a rate of 2.5 °C min^−1^ to reach a final temperature of 220 °C where it was held for 5 min. The injector was isothermal at 250 °C and split injection, 1:100, was used. The detector was held at 270 °C and Ar was employed as make-up gas to improve sensitivity.

The following equation was used to determine concentration of squalene in an oil sample (mg squalene g^−1^), by comparison to a single reference standard:

$$C=\frac{A_{ref,23:0}}{A_{ref,sq}}\;\times \frac{M_{ref,sq}}{M_{ref,23:0}}\times \frac{A_{sample,sq}}{A_{sample,23:0}}\times \frac{M_{sample,23:0}}{M_{oil}}\times 1000$$

where A_ref,23:0_ is the peak area of 23:0 in the reference standard, A_ref,sq_ is the peak area of squalene in the reference standard, M_ref,sq_ is the mass of squalene in the reference standard, M_ref,23:0_ is the mass of 23:0 in the reference standard, A_sample,sq_ is the peak area of squalene in the sample, A_sample,23:0_ is the peak area of 23:0 in the sample, M_sample,23:0_ is the mass of 23:0 added to the reference standard, and M_oil_ is the mass of oil analyzed.

### Method validation

**Fig. 1:**
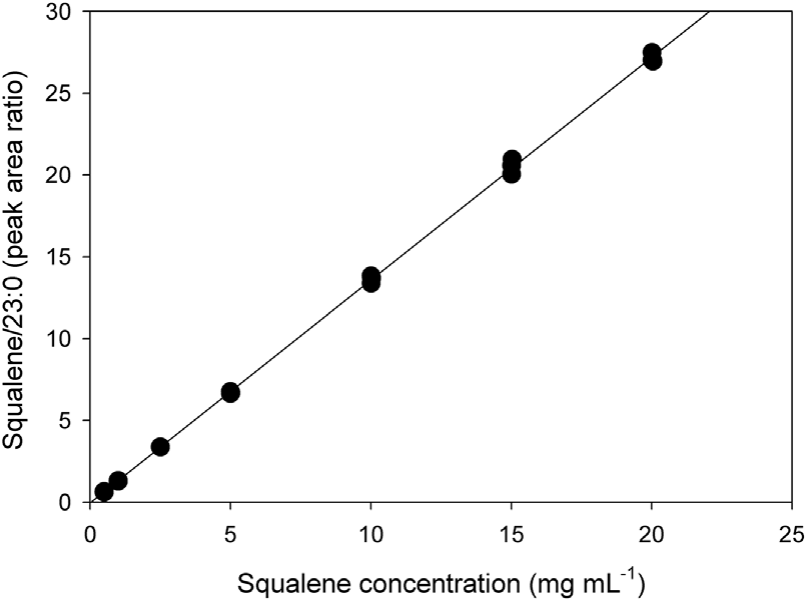
Calibration curve for squalene from 0.05 to 20 mg ml^−1^ (n = 21). The plot was linear with R^2^ = 0.9996. The equation of the line was y = 1.36x–0.03.

The calibration curve showed good linearity over the range 0.5–20 mg ml^−1^ (Fig. 1) with normally distributed residuals (Anderson-Darling test) and an insignificant lack of fit test. The regression equation was Y = 1.36X–0.03 with the intercept not significantly different than zero (Student’s T-test). Demonstration of this large linear range passing through zero allowed us to use a single reference standard for each subsequent determination, rather than developing a new standard curve for each analysis. Limits of detection (LOD) and quantification (LOQ) were determined using:

$$\text{L}\text{O}\text{D}\;=\;\frac{3s}{m}\;\text{a}\text{n}\text{d}\;\text{L}\text{O}\text{Q}=\;\frac{10s}{m}$$

where s is the standard error of the regression line and m is the slope. LOD and LOQ were 0.4 and 1.3 mg ml^−1^, respectively, setting a lower limit on squalene concentration in solution. This lower limit was easily avoided by setting ∼5 mg squalene ml^−1^ in hexane as the minimum concentration in all samples and standards. Tests of accuracy and precision were then carried out by running one reference standard of similar concentration to each sample and, in effect, for each batch of analyses, creating a two-point calibration curve, consisting of the standard concentration and zero.

Our tests of trueness with the standard addition experiments did not indicate a bias in results with respect to sample concentration (Table 1). Comparison of means (student t-test) indicated that there were no differences between true and experimental concentrations, with all relative differences <3%. We note the highest relative difference was found in the sample with the lowest squalene concentration that would be most influenced by weighing errors when creating the test material. Surrogate recoveries very near unity also indicated little bias.

**Table 1:** Results from standard addition experiments to evaluate accuracy. The actual concentration represents the known mass of squalene added to a known mass of fish oil (mean (sd); n = 3 for each concentration). The experimental concentration was determined using a single reference standard.

Actual Concentration (mg g^−1^)	Experimental Concentration (mg g^−1^)	Relative Difference (%)	Surrogate Recovery
5.01	5.15 (0.10)	2.77	1.03
39.55	39.48 (0.17)	−0.19	1.00
80.76	81.86 (0.32)	1.36	1.01

We were only able to test precision within this laboratory and thus refer to these tests as a measure of repeatability. Our data showed relative standard deviations that were ≤ the acceptable Horwitz [14] relative standard deviations and therefore indicated good repeatability (Table 2). These same tests of repeatability also allowed us to determine concentrations of squalene in natural oils. Both algal oils contained squalene concentrations at 3–5 mg g^−1^ oil, similar to that of olive oil (3 mg g^−1^). The squalene content in amaranth and shark liver oil were both considerably higher at 52 and 206 mg g^−1^, respectively.

**Table 2:** Experimentally determined concentrations (mean (sd); n = 5) of squalene in natural oils. Variation was assessed by comparison to the acceptable Horwitz relative standard deviation.

Sample	Concentration (mg g^−1^)	% RSD	Acceptable Horwitz % RSD
Algal Oil 1	4.79 (0.15)	3.2	3.2
Algal Oil 2	3.13 (0.05)	1.6	3.5
Olive Oil	2.88 (0.08)	2.7	3.3
Amaranth Oil	52.17 (0.10)	0.20	4.8
Shark Liver Oil	206.24 (0.51)	0.25	4.7

**Fig. 2:**
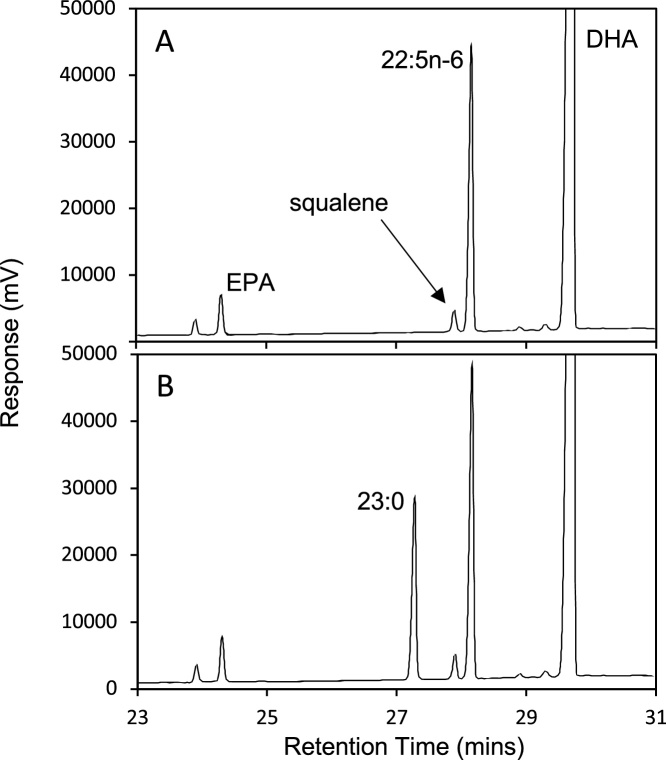
GCFID chromatogram showing resolution of FAME in an algal oil containing squalene without (A) and with (B) addition of 23:0 FAME as internal standard.

**Fig. 3:**
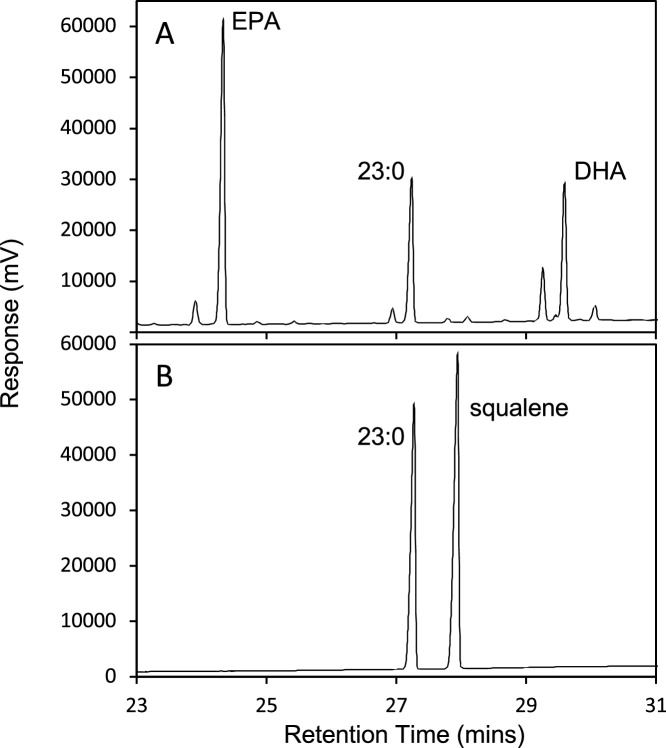
GCFID chromatogram showing resolution of (A) FAME in a fish oil without squalene; and (B) a reference standard containing 23:0 FAME as internal standard and squalene.

In summary, we developed and validated a simple method to determine squalene in edible and nutraceutical oils using a single reference standard. This rapid method requires ∼30 min for sample preparation and, although we did not demonstrate it in this analysis, this approach is easily adapted to also include the determination of EPA and DHA, shown in Fig. 2, Fig. 3. Such adaptation should include additional spiking and recovery tests to ensure that the conditions described here for squalene analysis are also appropriate for accurate determination of EPA ad DHA.

## Additional information

### GC resolution

Using the (50% cyanopropyl)-methylpolysiloxane phase and the temperature program given here, squalene eluted just before 22:5n-6 FAME, showing baseline resolution (Fig. 2). In fish oil, the minor fatty acid 22:4n-6 elutes just before squalene (Fig. 3), raising issues of partial co-elution; however, in the natural samples containing squalene, including the oils tested here, 22:4n-6 is either not present or present only in trace quantities and we did not attempt to manipulate resolution further. We also note that the internal standard, 23:0, is well resolved from all other FAME peaks and is not present in the algal oil (Fig. 2, Fig. 3). Thus, it is an appropriate choice as internal standard.

The 50%-cyanopropyl-methylpolysiloxane phase used here responds to changes in temperature much more than other polar columns; thus, increasing the oven temperature ramp may serve to elute squalene at earlier retention times relative to 22:4n-6. Modifying the column flow rate and/or using programmed flow rates will also influence resolution and should be evaluated if partial co-elution with 22:4n-6 becomes a problem.
